# ASD-FER: An Annotated Multi-Class Benchmark and Comparative Study for Facial Emotion Recognition in Children with Autism Spectrum Disorder

**DOI:** 10.64898/2026.10.14.754143

**Published:** 2026-10-20

**Authors:** Shahana Yasmin Chowdhury, Bithi Banik, Tracey Ann Knaus, Christopher David Moore, Henry Fordjour Ansah, Huda Hammad, Pranish Ghimire, Md Tamjidul Hoque, Shreya Banerjee

## Abstract

Autism Spectrum Disorder (ASD) affects social interaction and communication, and automatically recognizing emotions from the facial expressions of children with ASD remains challenging. A key barrier to *facial emotion recognition* (FER) in children with ASD is the absence of ASD-specific datasets with proper emotion-level annotations. To address this, we present **ASD-FER** , a benchmark of 2,779 facial images of children with ASD annotated across six emotion categories by three independent annotators using majority voting. We evaluate nine models — CNN, SVM, AlexNet, ResNet34, ResNet50, MobileNetV2, and three backbone configurations of our proposed task-specific architecture, **EQFormer** : ViT, Swin-T, and a compact Hybrid CNN-Transformer (HybridT) — under stratified 10-fold cross-validation and a held-out 90/10 evaluation. ResNet50 led conventional models at 84.89% mean cross-validation accuracy; among transformer-based models, ViT led with 88.27%, followed by Swin-T at 88.02% and HybridT at 86.58%. On the held-out set, ViT achieved the highest overall accuracy (91.01%), while Swin-T obtained the best balanced accuracy (74.47%) and macro-F1 (0.7078); HybridT remained competitive at 89.57% accuracy with a fraction of the parameters. To support explainability, Grad-CAM visualizations highlighted attention on emotion-relevant facial regions — eyes, eyebrows, and mouth — with Swin-T the most spatially focused and the HybridT the most parameter-efficient (2.66M). Class imbalance and cross-dataset generalization remain open challenges.

